# Supplementary material for: Development and Validation of the Interprofessional Collaboration Practice Competency Scale (IPCPCS) for Clinical Nurses
Source: Healthcare (Basel). 2024 Apr 8;12(7):806. doi: 10.3390/healthcare12070806 (PMC11012165; doi:10.3390/healthcare12070806)
Supplement: Supplementary file 1 [file healthcare-12-00806-s001.zip › healthcare-2713632-supplementary.pdf]

Supplemental

Table S1. IPCP instruments

Ref	Author (year)	Country	Questionnaire	Item	Scale type	Participants	Reliability	Construct validity	Doman (dimension)
	**Clincial Practitioners**								
12.	Baggs (1994)	USA	Collaboration and Satisfaction with Care Decisions (CSACD)	6+1	1(strongly disagree) to 7(strongly agree)	Neonatal intensive care nurses (n = 28) and pediatric residents (n = 26)	Cronbach's α: 0.95	EFA: PCA without rotation 1 factor with a total explained variance of 75.0%	NA
13.	Ushiro (2009)	Japan	Nurse-Physician Collaboration Scale (NPCS)	51→33	1(always) to 5(never)	Nurses (n = 1,217) and physicians (n = 446)	Cronbach's α: ≥ 0.8 for nurses and physicians<br>Test–retest reliability: ≥ 0.62 for nurses and physicians	1.EFA: PFM with a Promax rotation 3 factors<br>2.CFA: 3 factors for nurses and physicians	1.Sharing of patient information (9 items)<br>2.Joint participation in the cure/care decision-making process (12 items)<br>3.Cooperativeness (6 items)
14.	Kenaszchuk et al.(2010)	Canada	Adapted Nurses' Opinion Questionnaire (NOQ)	36→13	1(strongly disagree) to 4(strongly agree)<br>5 items: 4(strongly disagree) to 1(strongly agree)	Nurses, physicians and allied health professionals (n = 144)	Cronbach's α: 0.71 to 0.86 among nurses rating physicians, allied health professionals rating physicians or nurses	1.EFA: 3 factors with a Promax rotation<br>2.EFA/CFA: 3 factors<br>3.Full CFA: 3 factors	1.Communication (5 items)<br>2.Accommodation (5 items)<br>3.Isolation (3 items)
15.	Hull et al. (2011)	UK	Observational Teamwork Assessment for Surgery (OTAS)	130→114	0(problematic behavior; team function severely hindered) to 6(exemplary behavior; very highly effective in enhancing team function)	Phase 1: General surgical cases (n = 30)<br>Phase 2: OR experts (5 surgeons, 5 nurses, & 5 anesthesiologists) (n = 15)	ICC: Communication 0.77, coordination 0.67, cooperation 0.71, leadership 0.73, team monitoring<br>Interrater reliability: Surgeons 0.91, nurses 0.87, & anesthesiologists 0.91 (p < 0.001)	NA	1.Communication<br>2.Coordination<br>3.Cooperation / back up behavior<br>4.Leadership<br>5.Monitoring / situational awareness

Ref	Author (year)	Country	Questionnaire	Item	Scale type	Participants	Reliability	Construct validity	Doman (dimension)
16.	Shroder et al. (2011)	Canada	Collaborative Practice Assessment Tool (CPAT)	57→42→ 56	1(strongly disagree) to 7(strongly agree) + 3 open-ended questions	1.Practitioners (n = 42) including administration, medicine, nursing, nutrition services, OT, PT, social work, spiritual care, & volunteer service<br>2.Healthcare practitioners including RN, RPN, OT, physician, PT, etc. (n = 111)	1.Cronbach's α for 7 factors: 0.78, 0.81, 0.84, 0.73, 0.74, 0.73, & 0.74<br>2.Cronbach's α for 8 factors: 0.88, 0.89, 0.80, 0.81, 0.84, 0.76, 0.67, & 0.87	1.EFA: 7 factors<br>2.CFA: 8 factors	1.Mission, meaningful purpose, goals (8 items)<br>2.General relationships (8 items)<br>3.Team leadership (9 items)<br>4.General role responsibilities, autonomy (10 items)<br>5.Communication and information exchange (6 items)<br>6.Community linkages and coordination of care (6 items)<br>7.Decision-making and conflict management (4 items)<br>8.Patient involvement
17.	Sutton et al. (2011)	Austalia	Team Functioning Assessment Tool (TFAT)	11	7-point rating scale	Study 1: 8 professional groups (n = 63)<br>Study 2: experts (n = 6)<br>Study 3: interprofession (n = 10)	Inter-rater reliability: video 1(0.67 to 0.99), video 2(0.71 to 0.98), & video(0.82 to 0.98)	NA	1.Clinical planning (3 items)<br>2.Executive tasks (4 items)<br>3.Team relations (4 items)

Ref	Author (year)	Country	Questionnaire	Item	Scale type	Participants	Reliability	Construct validity	Doman (dimension)
18.	Orchard et al. (2012)	Canada	Assessment of Interprofessional Team Collaboration Scale (AITCS-II)	47→37	1(never) to 5(always)	Practitioners (n = 125) including RNs, physiotherapists, social workers, occupational therapists, pharmacists, physicians, dietitians, & practice nurses	Cronbach's α for each factor: 0.80 to 0.97<br>Cronbach's α for the overall scale: 0.98	1.EFA: PCA with a varimax rotation 3 factors with a total explained variance of 58.0%<br>2.CFA: a total explained variance of 60.4%	1.Partnership (19 items)<br>2.Cooperation (11 items)<br>3.Coordination (7 items)
19.	Van et al. (2012)	Australia	Pharmacist Frequency of Interprofessional Collaboration Instrument (FICI-P)	11→10	1(Nil) to 5(≥7 times)<br>1 to 4	Pharmacists (n = 224)	Cronbach's α: 0.90	1.EFA: PCA with an oblimin rotation 1 factor with a total explained variance of 52.7%<br>2.Rasch analysis: a moderate to good spread of items and thresholds	1 factor
20.	Van et al. (2012)	Australia	Frequency of Interprofessional Collaboration Instrument for GPs (FICI-GP)	11→10	1(Nil) to 5(≥7 times)<br>1 to 3	General practitioners (GPs) (n = 258)	Cronbach's α: 0.87	1.EFA: PCA with an oblimin rotation 1 factors with a total explained variance of 48.7%<br>2.Rasch analysis: a modest spread of items and thresholds	1 factor
21.	Jones et al.(2013)	USA	Jefferson Scale of Attitudes Toward Physician-Nurse Collaboration (JSATPNC)	15	13 items: 1(strongly disagree) to 4(strongly agree)<br>2 items: 4(strongly disagree) to 1(strongly agree)	Nurse practitioner (n = 915)	Cronbach's α for 3 factors: 0.61, 0.62, & 0.54.<br>Cronbach's α for the overall scale: 0.72	CFA: 3 factors	Factor 1(7 items)<br>Factor 2(6 items)<br>Factor 3(2 items)

Ref	Author (year)	Country	Questionnaire	Item	Scale type	Participants	Reliability	Construct validity	Doman (dimension)
22.	Yamamoto et al. (2014)	Japan	Interprofessional Collaboration (IPC) competeny	65→35	1(disagree) to 5(agree)	Health professionals including nursing profession, rehabilitation-related therapist, medical care-related profession, physician, dentist, pharmacists, dietician & registrant dietician, social welfare-related profession, clinical psychologists (n = 972)	Cronbach's α for 6 factors: 0.89, 0.83, 0.82, 0.69, 0.66, & 0.81	EFA: Promax rotation 6 factors	1. Respecting patients (10 items)<br>2. Team management skills (7 items)<br>3. Fulfilling one's role as a professional (5 items)<br>4. Attitudes and beliefs as a professional (4 items)<br>5. Attitudes that improve team cohesion (4 items)<br>6.Taken to achieve the team's goal (5 items)
23.	Tilden et al. (2016)	USA	Assessment for Collaborative Environments (ACE-15)	30→15	1(strongly disagree) to 4(strongly agree)	17 clinical professions and varied clinical setting (inpatient, ambulatory, urban, & rural) (n = 192)	Cronbach's α for the overall scale: 0.91	EFA: An oblimin rotation 1 factor with a total explained variance of 45.0%	NA
24.	Jadotte et al. (2017)	USA	Hospital Consummer Assessment of Healthcare Providers and Systems (HCAHPS) survey	32	NA	NA	NA	NA	1.Value / ethics for interprofessional practice (14 items)<br>2.Interprofessional communication (7 items)<br>3.Teams and teamwork (7 items)<br>4.Roles / responsibilities (4 items)

Ref	Author (year)	Country	Questionnaire	Item	Scale type	Participants	Reliability	Construct validity	Doman (dimension)
25.	Sakai et al. (2017)	Japan	Chiba Interprofessional Competency Scale (CICS29)	35→40→ 29	1(disagree) to 5(agree)	Study A: Nursing professional, rehabilitation-related therapist, medical care-related professional, physician, pharmacist, social welfare-related professional, dietician & registrant dietician, & clinical psychologists (n = 1,245)<br>Study B: RNs, RPNs, public health nurse, & midwife (n = 178)	Cronbach α for 6 factors: 0.89, 0.86, 0.91, 0.82, 0.84, & 0.83	CFA: 6 factors	1.Attitude and beliefs as a professional (6 items)<br>2.Team management skills (5 items)<br>3.Actions for accomplishing team goals (5 items)<br>4.Providing care that respects patients (5 items)<br>5.Attitudes and behaviours that improve team cohes (4 items)<br>6.Fulfilling one's role as a professional (4 items)
26.	Shimmura & Tadaka (2017)	Japan	Interprofessional Collaboration Competency Scale for Children with Medical Complexity (ICC-CMC)	30→12	0(disagree) to 3(agree)	Interprofessionals including physician, nurse, public health nurse, midwife, social worker, psychiatric social work, physiotherapist, occupational therapist, nursery teacher, school nurse, and others (n = 378)	Cronbach's α for 3 factors: 0.928, 0.885, & 0.893<br>Cronbach's α for the overall scale: 0.933	EFA: PFA with a Promax rotation 3 factors with a total explained variance of 72.55%<br>CFA: 3 factors	1.Sharing needs assessment skills (4 items)<br>2.Resource development skills (4 items)<br>3.Creative networking skills (4 items)
27.	Meijer et al. (2018)	Netherlands	Interprofessional -Interorganisational Collaboration	10	1(none of the attribute) to 5(lots of the attribute)	GPs & SCCs (n = 445) from region 1(n = 203), 2(n = 84), & 3(n = 158)	Test-retest reliability for 10 items: Squared weighted Kappa 0.31 to 0.63 (insufficient for each item)	1.CFA: 2 factors<br>2.EFA: Oblimin rotation showed unstable factor	1.Interpersonal relationships: Shared goals and vision (2 items) &

Ref	Author (year)	Country	Questionnaire	Item	Scale type	Participants	Reliability	Construct validity	Doman (dimension)
			Questionnaire (IICQ)					structures across regions (region 1: 4 factors, region 2 & 3: 5 factors)	internalisation (2 items)<br>2.Organisational setting: Governance (4 items) & formalisation (2 items)
28.	Orchard et al. (2018)	Canada	Assessment of Interprofessional Team Collaboration Scale (AITCS-II)	37→23	1(strongly disagree) to 5(strongly agree)	Practitioners (n = 926 or 676)including RNs, RPNs, laboratory technicians, imaging technologists, physiologists, dietary aids, etc.	Cronbach α for 3 factors: 0.898, 0.924, & 0.898<br>Cronbach α for the overall scale: 0.894	1.EFA: PCA with a varimax rotation 3 factors with a total explained variance of 51.61%<br>2.CFA: 3 factors	1.Partnership (8 items)<br>2.Cooperation (8 items)<br>3.Coordination (7 items)
29.	Haruta & Goto (2022)	Japan	Japanese version of the Self-assessment Scale of Interprofessional Competency (JASSIC)	24→18	1 to 5	Healthcare professionals (n = 139 or 153) including visiting nurses, family physicians, occupational therapists, pharmacists, social workers, etc.	Cronbach's α for 6 factors: 0.95, 0.88, 0.87, 0.84, 0.90, & 0.81<br>Cronbach's α for the overall scale: 0.92<br>Pearson correlation between AITCS-II and JASSIC: 0.69	EFA: Promax rotation 6 factors with a total explained variance of 82.3%<br>CFA: 6 factors	NA
30.	Soemantri et al. (2022)	Indonesia	Indonesian CICS29	29	1(never) to 5(always)	Healthcare professionals including medicine, dentistry, public health, nursing, pharmacy, & other allied health professionals (n = 300)	Cronbach α for 6 factors: 0.732, 0.621, 0.726, 0.669, 0.657, & 0.656<br>Cronbach α for the overall scale: 0.921	CFA: 6 factors	1.Attitude and beliefs as a professional (6 items)<br>2.Team management skills (5 items)<br>3.Actions for accomplishing team goals (5 items)<br>4.Providing care that respects patients (5 items)

Ref	Author (year)	Country	Questionnaire	Item	Scale type	Participants	Reliability	Construct validity	Doman (dimension)
									5.Attitudes and behaviours that improve team cohes (4 items)<br>6.Fulfilling one's role as a professional (4 items)
31.	Hanskamp-Sebregts et al. (2023)	Netherlands	Interprofessional Collaborative Practice for Integrated Hospital care (IPPIH)	27	1(minimal collaboration) to 10(maximum collaboration)<br>1(poor) to 6(excellent) + cannot assess (-)	Step 3: Healthcare providers including medical specialists, nurse practitioners, paramedical staff, & secretarial assistants (n = 30)<br>Step 4: Healthcare providers including medical, nursing, paramedical, & others (n = 119)	Cronbach α for 5 factors: 0.91, 0.89, 0.87, 0.62, & 0.48<br>Cronbach α for the overall scale: 0.953	EFA: PCA with a varimax rotation 5 factors with a total explained variance of 66.7%	1.Own skills (8 items)<br>2.Culture (8 items)<br>3.Coordination and Collaboration (6 items)<br>4.Practical support (3 items)<br>5.Appreciation (2 items)

Ref	Author (year)	Country	Questionnaire	Item	Scale type	Participants	Reliability	Construct validity	Doman (dimension)
32.	Prasitanarapu & Kitreerawutiwong (2023)	Thailand	Interprofessional Collaboration (IPC) Competeny	49	1(lowest performing) to 5(highest performing)	Healthcare professionals (n = 37 or 497) including public health practitioners, nurses, Thai traditional medicine, physicians, dentists, pharmacists, & physiotherapists	Cronbach's α for 6 factors: 0.94, 0.93, 0.92, 0.90, 0.92, & 0.86<br>Cronbach's α for the overall scale: 0.97	1.EFA: PCA with a varimax rotation 6 factors with a total explained variance of 66.53%<br>2.CFA: 6 factors	1.Collaborative teamwork (13 items)<br>2.Population- and community-centred care (8 items)<br>3.Communication and mutual respect (10 items)<br>4.Clarification of roles and responsibilities (8 items)<br>5.Interprofessional reflection (6 items)<br>6.Interprofessional values and mixed skills (4 items)
	**Practicioners & students**								
33.	Archibald et al. (2014)	Canada	Interprofessional Collaborative Competency Attainment Survey (ICCAS)	20	1(strongly disagree) to 7(strongly agree) + 1 NA	Students and clinicians in 15 interprofessional education undergraduate, graduate, and continuing professional development programs (n = 584)	Cronbach's α for 2 factors: 0.961 & 0.941.<br>Cronbach's α for the overall scale: 0.981	EFA: PAF with an oblique oblimin rotation 2 factors for the pre-program with a total explained variance of 69.84% and for the post-program 1 factor with a total explained variance of 73.1%	1.One's own role and skills in collaboration (15 items)<br>2.One's involmement with the rest of the team/families (5 items)
	**Schools Students**								

Ref	Author (year)	Country	Questionnaire	Item	Scale type	Participants	Reliability	Construct validity	Doman (dimension)
34.	Hojat et al. (1999)	USA	Jefferson Scale of Attitudes Toward Physician Nurse Collaboration (JSAPNC)	15	13 items: 1(strongly disagree) to 4(strongly agree)<br>2 items: 4(strongly disagree) to 1(strongly agree)	1st year medical (n = 208) and nursing students in an upper-division baccalaureate program (n = 86)	Crnbach's α: 0.84 (medical) & 0.85 (nursing students)	EFA: PCA with a varimax rotation 4 factors with a total explained variance of 58%	1.Shared educational and collaborative relationships (7 items)<br>2.Caring as opposed to curing (3 items)<br>3.Nurse's autonomy (3 items)<br>4.Physician's authority (2 items)<br>5.Other (5 items)
35.	Ward et al. (2008)	USA	Jefferson Scale of Attitudes Toward Physician Nurse Collaboration (JSAPNC)	15	13 items: 1(strongly disagree) to 4(strongly agree)<br>2 items: 4(strongly disagree) to 1(strongly agree)	Undergraduate nursing students (n = 333)	Cronbach's α for 3 factors: 0.77, 0.66, & 0.57	EFA: PCA with a varimax rotation 3 factors with a total explained variance of 47.0%	1.Shared education and collaboration (7 items)<br>2. Caring and Nurse's autonomy (6 items)<br>3.Physician authority (2 items)
36.	Curran et al. (2011)	Canada	Interprofessional Collaborator Assessment Rubric (ICAR)	18	1(minimal) to 4(mastery)	1.Delphi survey experts (n = 21):<br>2.Delphi survey: experts (n = 12)<br>3.Focus groups with students (n = 5) and faculty (n = 7) at both college and university levels	NA	NA	1.Communication (2 items)<br>2.Collaboration (3 items)<br>3.Roles and responsibility (4 items)<br>4.Collaborative patient/ client –family centred approach (4 items)<br>5.Team functioning (3 items)<br>6.Confilict management / resolution (2 items)

Ref	Author (year)	Country	Questionnaire	Item	Scale type	Participants	Reliability	Construct validity	Doman (dimension)
37.	Chiu (2014)	USA	Performance Assessment Tools for Interprofessional Communication and Teamwork (PACT)	Novice: 5<br>Expert: 13<br>Video: 26 & global quality 5	1(poor) to 5(excellent)<br>Present, absent, or NA<br>1(poor) to 4(excellent)<br>1(need improvement in most areas) to 4(excellent)	Pilot study: Students from the schools of medicine, nursing, pharmach, & the PA (n = 49)<br>Interprofessional students including nursing, pharmacy, & PA (n = 306)	Cronbach's α for the overall scale: PACT-Novice 0.85; PACT-Expert 0.79; PACT-Video 0.91	EFA: PAF with a Varimax rotation 4 factors with a total explained variance of 62.76% for PACT-Expert and 6 factors with a total explained variance of 66.13% for PACT-Video	1.Team structure (2 /6 items)<br>2.Leadership (2 5 items)<br>3.Situation monitoring (2 / 4 items)<br>4.Mutual support (2 / 5 itmes)<br>5.Communication (5 / 6 items)
38.	Dow et al. (2014)	USA	Interprofessional Education Collaborative (IPEC) Competency Self-Assessment Tool	42	1(strongly disagree) to 5(strongly agree)	All the students on a health science campus include 5 health science schools – allied health, dentistry, medicine, nursing, & pharmacy (n = 481)	Cronbach's α for 4 factors: 0.96 to 0.98	EFA: PCA with a varimax rotation 4 factors with a total explained variance of 79.61%	1. Teams and teamwork (15 items)<br>2.Values and ethics (10 items)<br>3.Interprofessional communication (10 items)<br>4.Roles and responsibilities (7 items)
39.	Dominguez et al. (2015)	USA	Attitude Toward Health Care Teams (ATHCT-R)<br>Student Perceptions of Interprofessional Clinical Education-Revised (SPICE-R)	21<br>10	1(strongly disagree) to 6(strongly agree)<br>1(strongly disagree) to 5(strongly agree)	1st year Students in a variety of health professional degree programs including nursing, optometry, pharmacy, PT, & health administration (n = 221)	Cronbach's α for the overall scale: 0.79 for ATHCT-R & 0.86 for SPICE-R	CFA: SPICE-R better goodness of fit, construct	ATHCT-R<br>1.Team value (11 items)<br>2.Team efficiency (5 items)<br>3.Shared leadership (5 items)<br>SPICE-R<br>1.Outcomes (2 items)<br>2.Team work (6 items)<br>3.Roles / responsibility (2 items)

Ref	Author (year)	Country	Questionnaire	Item	Scale type	Participants	Reliability	Construct validity	Doman (dimension)
40.	Lockeman et al. (2016)	USA	Interprofessional Education Collaborative (IPEC) Competency Self-Assessment Tool revised	16	1(Strongly Disagree) to 5(Strongly Agree)	Study 1: Diverse health professions students at a single institution (n = 450)<br>Study 2: Health professional students across 4 institutions<br>Study 3: Combine 1 & 2 (n = 547)	1.Cronbach's α for 3 factors: >0.97<br>2.Cronbach's α for 3 factors: >0.96<br>3.Cronbach's α for 2 factors: 0.92 & 0.96	1.EFA: ML vs. PAF with a varimax vs. direct oblimin rotation 3 factors<br>2.EFA: ML vs.PAF with a varimax vs. direct oblimin rotation 3 factors<br>3.CFA: 2 factors with adequate model fit	1.Interprofessional interaction (7 items)<br>2.Interprofessional values (9 items)
41.	Hasnain et al. (2017)	USA	Interprofessional Education Collaborative Competence Self-Efficacy Tool (IPECC-SET-38)	38→27	100-mm VAS → recoded into 10 category scores: 0–9	Health professions students (n = 658)	Cronbach's α for 4 factors: 0.92 to 0.94	PCA: 2 factors<br>Rasch analysis: 4 factors fit to the Rasch model	1. Teams and teamwork (11 items)<br>2.Values and ethics (10 items)<br>3.Interprofessional communication (8 items)<br>4.Roles and responsibilities (9 items)
42.	Schmitz et al. (2017)	Canada	Interprofessional Collaborative Competency Attainment Survey (ICCAS) revised	20	1(poor) to 5(excellent)	Medicine, pharmacy, dentistry, veterinarian medicine, public health, nursing, other programmes' students (n = 785 & 584)	Cronbach's α for the overall scale: 0.96	EFA: PCA with a varimax rotation 1 factor with a total explained variance of 85%	1 factor

Ref	Author (year)	Country	Questionnaire	Item	Scale type	Participants	Reliability	Construct validity	Doman (dimension)
43.	Edelbring et al. (2018)	Sweden	Jefferson Scale of Attitudes Towards Physician-Nurse Collaboration (JSAPNC)<br>Readiness for Interprofessional Learning Scale (RIPLS)	1.15<br>2.19	JSAPNC: 13 items: 1(strongly disagree) to 4(strongly agree) & 2 items: 4(strongly disagree) to 1(strongly agree)<br>RIPLS: 1(strongly disagree) to 5(strongly agree)	JSAPNC including nursing & medical students (n = 88)<br>RIPLS including nursing & medical students (n = 84)	JSAPNC: Cronbach's α for 4 factors: 0.46, 0.44, 0.40, 0.63 & the overall scale 0.62<br>RIPLS: Cronbach's α for 3 factors: 0.79, 0.75, 0.36 & the overall scale 0.66	NA	JSAPNC<br>1.Shared educational Experiences and Professional Collaboration (7 items)<br>2.Caring as opposed to curing (3 items)<br>3.Nurse's autonomy (3 items)<br>4.Physician's authority (2 items)<br>RIPLS<br>1.Teamwork and collaboration (9 items)<br>2.Professional identity (7 items)<br>3.Roles and responsibilities (3 items)
44.	Hinyard et al. (2018)	USA	Self-Assessment of Collaboration Skills (SACS)	35→19→11	1(strongly disagree) to 7(strongly agree)	Undergraduate health professions students<br>1.Pilot test 1 (n = 160)<br>2.Pilot test 2 (n = 131)<br>3.Pilot test 3 (n = 181)	1.Cronbach's α for 3 factors: 0.74, 0.83, & 0.93<br>2.Cronbach's α for 3 factors: 0.77, 0.84, & 0.87<br>3.Cronbach's α for 3 factors: 0.67, 0.84, & 0.86	1.EFA: Promax rotation 3 factors with a total explained variance of 69.79%<br>2.EFA: Promax rotation 3 factors with a total explained variance of 63.84%<br>3.CFA: 3 factors	1.Information sharing (2 items)<br>2.Team support. (3 items)<br>3.Learning (6 items)

Ref	Author (year)	Country	Questionnaire	Item	Scale type	Participants	Reliability	Construct validity	Doman (dimension)
45.	Kottorp et al. (2019)	USA	Interprofessional Education Collaborative Competence Self-Efficacy Tool (IPECC-SET-9)	38→27→ 9	100-mm VAS a → recoded into 10 category scores: 0–9	2 cohorts of students from 11 health professions programs (n = 658 & 696)	Cronbach α for the overall scale: 0.98 (27 items) Cronbach α for the overall scale: 0.94 (9 items)	Rasch analysis EFA: PCA 27 items 61.2% explained variance & 9 items 64.2% explained variance	Perceived competence in interprofessional collaboration (9 items)
46.	Violato & King (2019)	Canada	Interprofessional Collaborative Competency Attainment Survey (ICCAS)	20	1(poor) to 5(excellent) + 1 NA	Health professional students (n = 991)	Cronbach's α for the pre-test & post-test: 0.97 & 0.95	EFA: post-test data varimax rotation 1 factor	1 factor
47.	Braathen (2022)	Norway	Self-Assessment of Collaboration Skills (SACS)	11	1(strongly disagree) to 7(strongly agree)	Nursing, social work, teacher education, physiotherapy, OT, child welfare services, & kindergarten teacher students (n = 499)	Cronbach α for 3 factors: 0.73, 0.78, & 0.58	EFA: PAF with an oblique rotation 3 factors with a total explained variance of 60.87%	1.Learning (5 items) 2.Team support (4 items) 3.Information sharing (2 items)

Note. EFA, Exploratory Factor Analysis; PCA, Principal Component Analysis; PFA, Principal Factor Analysis; PAF, Principal Axis Factoring; ML, Masimum Likelihood; CFA, Confirmative Factor Analysis; ICC, Intraclass Correlation; RNs, Registered Nurses; RPNs, Registered Practice Nurses; MDs, Doctors of Medicine; GPs, General Practitioners; SCCs, Secondary Care Clinicians; OT, Occupational Therapy; PT, Physical Therapy; PA, Physician Assistant; Visual Analogue Scale, VAS; NA, Not Applicable

Supplemental

Table S2. IPCP six competency domains and definitions vs. IPCPCS three domains.

Domains	Competency statement	Definition	IPCPCS new domains
Collaborative leadership	Learners/practitioners understand and can apply leadership principles that support a collaborative practice model. this domain supports shared decision-making as well as leadership but it also implies continued individual accountability for one's own actions, responsibilities and roles as explicitly defined within one's professional/disciplinary scope of practice.	Involves guiding and coordinating a group of professionals from different disciplines to work together effectively. This leadership style is characterized by shared decision-making, mutual respect, and a focus on achieving the best patient outcomes.	Factor 1: Collaborative Leadership and Interprofessional Conflict Resolution (CLICR)
Interprofessional conflict resolution	Learners/practitioners actively engage self and others, including the client/patient/family, in positively and constructively addressing disagreements as they arise.	The capacity to acknowledge, address, and navigate interpersonal and interprofessional conflicts in a constructive and collaborative manner. It includes recognizing potential conflicts, employing strategies to resolve them, and maintaining a professional and respectful environment.	
Interprofessional communication	Learners/ practitioners from different professions communicate with each other in a collaborative, responsive and responsible manner.	The ability to exchange information, ideas, and feelings among team members from different professional backgrounds in a collaborative, responsive, and responsible manner. It emphasizes the importance of clear, respectful, and purposeful communication in interprofessional settings.	Factor 2: Interprofessional Communication and Team Functioning (ICTF)
Team functioning	Learners/practitioners understand the principles of team work dynamics and group/team processes to enable effective interprofessional collaboration.	This domain relates to the ability to effectively operate within an interprofessional team. It includes understanding team dynamics, contributing to a positive team environment, and facilitating collaborative decision-making and problem-solving.	
Patient/client/family /community-centred care	Learners/practitioners seek out, integrate and value, as a partner, the input, and the engagement of the patient/client/family/community in designing and implementing care/services.<br>a partnership between a team of health providers and a patient where the patient retains control over his/her	This domain focuses on delivering care that is respectful of, and responsive to, the preferences, needs, and values of patients/clients and their families or communities. It underscores the importance of involving them as active partners in the healthcare process.	Factor 3: Role Clarification and Client-Centred Care (RCCC)

Domains	Competency statement	Definition	IPCPCS new domains
	care and is provided access to the knowledge and skills of team members to arrive at a realistic team shared plan of care and access to the resources to achieve the plan		
Role clarification	Learners/practitioners understand their own role and the roles of those in other professions, and use this knowledge appropriately to establish and achieve patient/client/family and community goals.	Involves understanding and articulating one's professional role and the roles of other team members. It emphasizes the importance of acknowledging and respecting the diverse roles, responsibilities, and competencies within the healthcare team to optimize collaborative practice.	

Reference: [5,11]
